# Supplementary material for: Adjunctive Procedures in Immediate Implant Placement: Necessity or Option? A Systematic Review and Meta-Analysis
Source: Materials (Basel). 2025 Dec 2;18(23):5427. doi: 10.3390/ma18235427 (PMC12693628; doi:10.3390/ma18235427)
Supplement: Supplementary file 1 [file materials-18-05427-s001.zip › materials-3974649-supplementary.pdf]

Supplementary Table S1. Reasons for exclusion.

Reason for exclusion	Excluded studies
Absence of a “non-adjunctive” control group	Prosper et al. (2003); Hassan et al. (2008); De Angelis et al. (2011); Pieri et al. (2011); Spinato et al. (2012); Viswambaran et al. (2014); Migliorati et al. (2015); Stoupel et al. (2016); ArRejaie et al. (2016); Hazzaa et al. (2017); Mastrangelo et al. (2018); Bittner et al. (2019); Chan et al. (2019); Fettouh et al. (2020); Shahid et al. (2021); Atef et al. (2021); Garcia-Sanchez et al. (2021); Tomar et al. (2022); Elaskary et al. (2022); Elbrashy et al. (2022); Abdullah et al. (2022); Wanis et al. (2022); Borgia et al. (2022); Puisys et al. (2022); Happe et al. (2022); ElAmrousy et al. (2022); Elshikh et al. (2023); Lee et al. (2023); Hamed et al. (2023); Fernandes et al. (2023); Soni et al. (2023); Khalifah et al. (2023); Azaripour et al. (2023); Rani et al. (2024); Purohit et al. (2024); Cosyn et al. (2024); Zamora et al. (2024); Abo-elkheir et al. (2024); Ellithy et al. (2024); Rasheed Issa et al. (2024); Elaskary et al. (2024); Fettouh et al. (2024); Gurbuz et al. (2025); El-Danasory et al. (2025)
Insufficient follow-up	Paknejad et al. (2017); Sayin et al. (2022); Iram et al. (2024)
Wrong comparator	Cordaro et al. (2009); Koh et al. (2011); Cecchinato et al. (2013); Grandi et al. (2014); Zafiropoulos et al. (2017); Peñarrocha-Oltra et al. (2018); Clementini et al. (2019); Slagter et al. (2021)
Non-relevant outcomes	Rungcharassaeng et al. (2012); Peñarrocha-Oltra et al. (2018)
Implant site not corresponding to inclusion criteria	Shibly et al. (2010); Diana et al. (2018); Chen et al. (2005); Chen et al. (2007); Yuenvongorarn et al. (2020); Vivek et al. (2024); Rasheed Issa et al. (2024b)
Ineligible study design	Cranin et al. (1988); Rebaudi et al. (2003); Cornelini et al. (2008); Crespi et al. (2008); Wu et al. (2019); Cardaropoli et al. (2019)
Insufficient sample size	Tiwari et al. (2020); Purohit et al. (2024)

Figure S1. Funnel Plot.

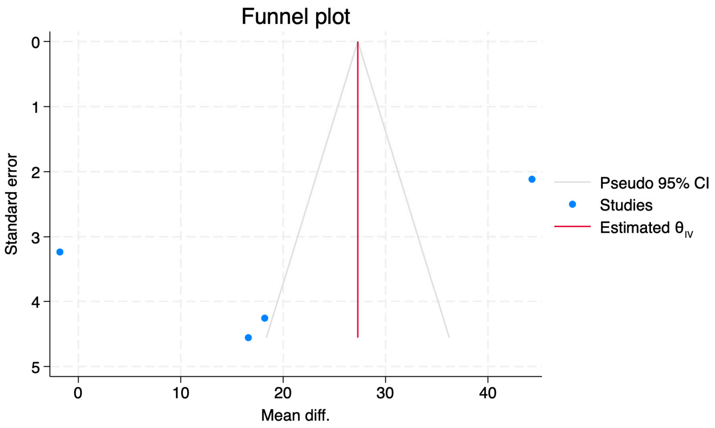

Appendix SA1

Search strings utilized for electronic database search:

PubMed:

(((dental implant OR implant OR Fixture OR Type I protocol) AND (postextraction OR fresh extraction sockets OR immediate implant OR dehiscence) AND (graft OR grafting OR filling material OR bone substitute biomaterial OR Bone OR Biomaterial OR Sub-epithelial connective tissue graft OR Soft tissue substitute) AND (RCT OR randomized controlled trial OR random OR Controlled Clinical Trial)))*

Scopus:

(dental implant) OR (implant) OR (Fixture) OR (Type I protocol) AND (post-extraction) OR (fresh extraction sockets) OR (immediate implant) OR (dehiscence) AND (graft) OR (grafting) OR (filling material) OR (bone substitute biomaterial) OR (Bone) OR (Biomaterial) OR (Sub-epithelial connective tissue graft) OR (Soft tissue substitute) AND (RCT) OR (randomized controlled trial) OR (random) OR (Controlled Clinical Trial)

EMBASE:

('dental implant'/exp OR 'implant'/exp OR 'fixture' OR 'type i protocol') AND ('postextraction' OR 'fresh extraction sockets' OR 'immediate implant' OR 'dehiscence'/exp) AND ('graft'/exp OR 'grafting'/exp OR 'filling material' OR 'bone substitute biomaterial' OR 'bone'/exp OR 'biomaterial'/exp OR 'sub-epithelial connective tissue graft' OR 'soft tissue substitute') AND ('randomized controlled trial'/exp OR 'randomized controlled trial' OR random OR 'controlled clinical trial'/exp)*
